# Human extracellular vesicles and correlation with two clinical forms of toxoplasmosis

**DOI:** 10.1371/journal.pone.0229602

**Published:** 2020-03-03

**Authors:** Allecineia Bispo da Cruz, Marta Marques Maia, Ingrid de Siqueira Pereira, Noemi Nosomi Taniwaki, Gislene Mitsue Namiyama, João Paulo Marochi Telles, Jose Ernesto Vidal, Lígia Cosentino Junqueira Franco Spegiorin, Cinara Cássia Brandão de Mattos, Luiz Carlos de Mattos, Cristina da Silva Meira-Strejevitch, Vera Lucia Pereira-Chioccola

**Affiliations:** 1 Centro de Parasitologia e Micologia, Instituto Adolfo Lutz, Sao Paulo, Brazil; 2 Nucleo de Microscopia Eletrônica, Instituto Adolfo Lutz, Sao Paulo, Brazil; 3 Instituto de Infectologia Emilio Ribas, São Paulo, Brazil; 4 Faculdade de Medicina, Hospital das Clínicas, da Universidade de São Paulo, São Paulo, Brazil; 5 Laboratório de Investigação Médica (LIM) 49, Instituto de Medicina Tropical da Universidade de São Paulo, São Paulo, Brazil; 6 Hospital de Base, São José do Rio Preto, Brazil; 7 Faculdade de Medicina de São José do Rio Preto, São José do Rio Preto, Brazil; Institut national de la santé et de la recherche médicale - Institut Cochin, FRANCE

## Abstract

**Aim:**

This study analyzed microvesicles and exosomes, called as extracellular vesicles (EVs) excreted in serum and cerebrospinal fluid (CSF) from patients with cerebral or gestational toxoplasmosis.

**Methods:**

Clinical samples from 83 individuals were divided into four groups. Group I, 20 sera from healthy individuals and pregnant women (seronegative for toxoplasmosis); group II, 21 sera from seropositive patients for toxoplasmosis (cerebral or gestational forms); group III, 26 CSF samples from patients with cerebral toxoplasmosis/HIV co-infection (CT/HIV) (seropositive for toxoplasmosis); and group IV, 16 CSF samples from seronegative patients for toxoplasmosis, but with HIV infection and other opportunistic infections (OI/HIV). Serum and CSF samples were ultracentrifuged to recover EVs. Next, vesicle size and concentration were characterized by Nanoparticle Tracking Analysis (NTA).

**Results:**

Concentrations of serum-derived EVs from toxoplasmosis patients (mean: 2.4 x 10^10^ EVs/mL) were statically higher than of non-infected individuals (mean: 5.9 x 10^9^ EVs/mL). Concentrations of CSF-derived EVs were almost similar in both groups. CT/HIV (mean: 2.9 x 10^9^ EVs/mL) and OI/HIV (mean: 4.8 x 10^9^ EVs/mL). Analyses by NTA confirmed that CSF-derived EVs and serum-derived EVs had size and shape similar to microvesicles and exosomes. The mean size of EVs was similar in serum and CSF. Thus, the concentration, and not size was able distinguish patients with toxoplasmosis than healthy individuals. Presence of exosomes was also confirmed by transmission electron microscopy and evidence of tetraspanins CD63 and CD9 in immunoblotting. Relative expressions of miR-146a-5p, miR-155-5p, miR-21-5p, miR-29c-3p and miR-125b-5p were estimated in exosomal miRNA extracted of EVs.

Serum-derived EVs from group II (cerebral and gestational toxoplasmosis) up-expressed miR-125b-5p and miR-146a-5p. CSF-derived EVs from CT/HIV patients) up-expressed miR-155-5p and miR-21-5p and were unable to express miR-29c-3p.

**Conclusion:**

These data suggest the participation of EVs and exosomal miRNAs in unbalance of immune response as elevation of TNF-α, IL-6; and downregulation of IFN-γ in cerebral and gestational forms of toxoplasmosis.

## Introduction

Toxoplasmosis, caused by *Toxoplasma gondii* is an infection asymptomatic in immunocompetent hosts. Chronic phase is characterized by persistence of encysted parasites in brain and muscle. In asymptomatic or subclinical form, mild symptoms occur during first few weeks [1–3]. Nevertheless, symptomatic forms may occur in 10–20% and complications such as, acute respiratory failure or shock. Thus, toxoplasmosis could be a serious public health problem, as it may lead to more severe symptoms [4,5]. Infections occurring during pregnancy, may led to neonatal malformations and ocular difficulties in fetus [6–8]. Ocular forms are characterized by necrotic lesions and are result from congenital or after birth-acquired infections. These lesions may destroy neural retina architecture and sometimes choroid (retinochoroiditis) is affected [9,10]. Reactivation of latent infection in immunodeficiency, such as people living with HIV and without combination antiretroviral therapy, results, in cerebral toxoplasmosis. In rare cases, due to failure of Th1 immune response disseminated toxoplasmosis might occur, which involves at least two organs [5,11–14].

*T*. *gondii* induces changes in immune response of infected hosts in both, innate and adaptive ones. Thus, infected hosts develop life-long protective immunity against re-infection against. Consequently, antigen secretion is essential in boosting the immune system since they stimulate the T and B-cell responses [4,15]. These studies have been shown that development of the clinical forms of toxoplasmosis is correlated with the different aspects of the relationship between parasite and infected host [5,16–18].

Extracellular vesicles (EVs) are a group of small structures that are released by cells [19]. EVs are produced by most cell types and play an important role in cell-cell communication. Among different functions, these nano-particles can delivery various cargos as proteins, lipids, DNA, RNA, including micro-RNA (miRNA); and have different secretory components under physiological or pathological, conditions [20,21]. EVs transfer biomarker and macromolecules in specific diseases, contributing to disease pathogenesis [21–23].

EVs are purified by ultracentrifugation or chromatography. Among methods for examination include the electron microscopy and Nanoparticle Tracking Analysis (NTA) [19]. EVs comprise a variety of membrane-limited vesicles released from cells, and are classified into three subclasses, based on their origin or size. The subclass exosomes are the smallest and best characterized vesicles (30–100 nm). They do not originate from plasma membrane, but they are derived from internal budding of vesicles in the lumen of early endosome. Exosomes contain different set of proteins such as the Alix, TSG101, HSP70 and the tetraspanins CD63, CD81 and CD9 [24]. The microvesicles vary in size from 100 to 1,000 nm and are produced by external budding from the plasma membrane. Finally, the apoptotic bodies represent the largest EVs with size ranging from 1,000 to 5,000 nm; the name is due to their origin, since they are released as blebs from cells undergoing programmed death cell [22,24–28].

The participation of EVs in interaction between *T*. *gondii* and hosts is not yet established. Regarding on *T*. *gondii*, proteomic profile of exosomes containing a wide range of proteins [29]. *T*. *gondii* derived-EVs (exosomes and microvesicles) contain miRNA and they were immunologically recognized by host immune response inducing humoral and cellular responses as IL-10, TNF-α, and iNOS [30,31]. Exosomes excreted by infected host cells constituted an effective non-cellular vaccine with antigen and adjuvant properties for the immune response as well as, immunization [32,33]. *T*. *gondii* infection alters cell proliferation mechanisms of infected cells and exosomes secreted by *T*. *gondii*-infected cells can mediate such changes to neighboring cells [34–36]. However, the interaction between *T*. *gondii* infection and EVs derived of humans is unknown. Thus, this study was aimed to characterize these nanoparticles, including microvesicles and exosomes excreted in serum and cerebrospinal fluid (CSF) from patients with cerebral or gestational toxoplasmosis.

## Materials and methods

### Ethical statements

The Ethic Committees of Instituto Adolfo Lutz (CONEP-IAL/SES number: 2922263), Instituto Emilio Ribas (CONEP-IIER number: 1133380) and Faculdade de Medicina de São José do Rio Preto (CEP-FAMERP number: 712142) by Plataforma Brasil (from Brazilian Ministry) approved this study, with waive of the patient consent, which was performed according recommendations of Plataforma Brasil.

### Clinical samples

This study analyzed human clinical samples from 83 individuals (41 serum samples and 42 CSF) divided into four groups.

Group I was formed of 20 serum samples from healthy individuals and pregnant women. All of them were seronegative for toxoplasmosis in ELISA. In period of blood collection, none of them was in treatment for any infection or disease.

Group II was formed of 21 serum samples from seropositive patients for toxoplasmosis (gestational or cerebral forms). Clinical diagnosis was established by clinical manifestations, and laboratorial, by ELISA, and qPCR. HIV-infected patients were admitted and treated at Instituto de Infectologia Emilio Ribas, in São Paulo, Brazil. Clinical diagnosis of cerebral toxoplasmosis in HIV-infected patients was based on: 1) progressive neurological deficits; 2) contrast-enhancing mass lesion(s) on computed tomography and/or magnetic resonance imaging; and 3) successful clinical and radiological response to antiparasitic treatment within 10–14 days [13,14]. Pregnant women with suspicion of acute toxoplasmosis infection were admitted and treated at the High-Risk Antenatal Care and Fetal Medicine Service (Hospital de Base, Sao José do Rio Preto, Brazil) After enrolling in a high-risk pregnancy clinic, pregnant women were routinely screened for TORSCH (Toxoplasmosis, Rubella, Syphilis, Cytomegalovirus, Hepatitis and HIV) [37,38] Those clinically, epidemiologically suspected of toxoplasmosis and having positive IgM or low IgG avidity for anti-*T*. *gondii* antibodies were underwent to amniocentesis and peripheral blood for PCR. Before collection pregnant women were informed of the procedure and signing informed-consent forms.

Group III was composed of 26 CSF samples from patients with cerebral toxoplasmosis/HIV co-infection (CT/HIV). The diagnosis was determined by clinical manifestations, ELISA, and qPCR, as described in Group II item.

Group IV was formed of 16 CSF samples from seronegative patients for toxoplasmosis, but with HIV infection and other opportunistic infections (OI/HIV). These HIV-infected patients, also, were admitted and treated at Instituto de Infectologia Emilio Ribas, in São Paulo, Brazil. Negative diagnosis for toxoplasmosis was evaluated by ELISA, while the other opportunistic infections, by clinical, laboratorial and radiological features.

All clinical samples (CSF, 3 mL and blood, 5 mL) were sent to laboratory within 48 hours after collection and immediately processed.

### Laboratorial diagnosis for toxoplasmosis

The laboratorial diagnosis for toxoplasmosis in the 83 clinical samples was evaluated by ELISA using a tachyzoite lysate antigen (TLA) as described before [39,40]. In samples from patients with cerebral or gestational, qPCR was performed using the REP-529 molecular marker, which amplifies a highly repeatable 112 bp sequence in *T*. *gondii* genome [41].

### Human EV purification by ultracentrifugation

CSF (600 μL) and serum (300 μL) samples were centrifuged at 13,500 x g for 15 minutes, to remove pellets, containing dead cells and debris. The supernatants containing 250 μL and 500 μL of serum and CSF, respectively, were transferred into Ultra-Clear Centrifuge tubes (6 mL tube for SW-55 rotor), (Beckman Coulter, Brea, CA, USA) and the volume completed until 6 mL with filtered phosphate-buffered saline (PBS), pH 7.2. The samples were ultracentrifuged at 100,000 x g for 60 minutes at 25° C in a Beckman® Coulter L8-80M ultracentrifuge. The pellets, containing EVs were resuspended in 100 μL of filtered PBS and stored at—20 °C until analysis.

### Nanoparticle Tracking Analysis (NTA)

After ultracentrifugation, concentration (particles/mL) and particle size (nm) CSF- derived EVs and serum-derived EVs were evaluated by nanoparticle tracking analysis (NTA) using the NanoSight NS300 instrument (Malvern—NanoSight^™^, NTA 3.0). NanoSITE-NTA computes size and number of particles based on their measured Brownian motion. Capturing and analyzing settings were done according to the equipment protocol. The analyses of concentration and size were performed with coefficient and hydrodynamic radius that were determined using the Stokes–Einstein equation, produced in NanoSight NS300 instrument. Results displayed as a particle size distribution. Data were presented as the average and standard deviation of three video recordings of 30–60 seconds per sample. Since NTA is accurate between particle concentrations in the range of 2 x 10^7^ to 2 x 10^9^/mL, EV samples were diluted before analysis in filtered PBS and the relative concentration calculated according to the dilution factor.

### Transmission electron microscopy (TEM)

Human EVs were previously fixed in 2% paraformaldehyde/PBS (v:v) for 1 hour. One drop of suspension was put on EM grid and performed by negative staining technique with 2% potassium phosphotungstate at pH 6,8, as previously described [31]. Grids were observed under a JEOL Transmission Electron Microscope (model JEM1011) (JEOL/Massachusetts/USA) operating at 80 kV. Images were recorded with a Gatan 785 ES1000W Erlangshen camera.

### EVs and immunoblotting

Serum-derived EVs and CSF-derived EVs were solubilized in lysis buffer (2% SDS, 10% glycerol, 5% 2-mercaptoethanol, 60 mM Tris-HCl, pH 6.8 and 0.002% bromophenol blue), boiled and run in 10% polyacrylamide-SDS gels. The investigation of EV proteins was done after silver staining. With the intention to determine exosomes in purified EVs by immunoblotting, EV proteins were transferred to nitrocellulose membranes. After blocked (for 60 minutes with 5% skim milk-PBS), membranes were incubated with anti-CD63 and anti-CD9 antibodies (Invitrogen®) diluted 1: 500 at 4° C for 18 hours. These antibodies recognize specifically the endosome-specific tetraspanins CD9 and CD63 (exosomal membranes). After washes, the membranes were incubated for 1 hour at room temperature with a goat horseradish peroxidase-conjugated anti-mouse IgG diluted (1:500) (Sigma®) in 5% skim milk-PBS. Bound antibodies were visualized using the quimioluminescence western blotting substrate (Pierce ECL Western Solution, Thermo Scientific®) and registered in Blot Scanner (C-DiGit®) according to the manufacturer’s instructions.

### RNA/miRNA isolation and cDNA synthesis

Total RNA including miRNA were extracted from serum-derived EVs (100μL). and CSF-derived EVs (100μL). Previously, to minimize RNA degradation by RNases during the experimental process, all materials and working surfaces were cleaned using RNaseZap® RNase Decontamination Solution (AmbionTM) prior to handling the samples. Purifications were performed using the miRNeasy Mini Kit (Qiagen), according to manufacturing instructions. EVs were mixed with a denaturing buffer in volumes described in the manufacture’s protocols. The homogenate was incubated at room temperature for 5 minutes. As internal control for extractions, 25 fmol of synthetic *Caenorhabditis elegans* miRNA (Cel-miR-39, Ambion) were spiked to each sample (Mitchel et al., 2008). RNA pellets were dissolved in DEPEC (RNase-free) water (30 μL). Next, 2 μL RNA including miRNA were reverse-transcripted (RT) using the Taqman®Advanced miRNA cDNA Synthesis kit (Life Technologies) according to manufacturing instructions. The reactions were performed in a Veriti® 96-Well Thermal Cycler (Applied Biosystems®) following four steps under the following thermal conditions: 45 minutes at 37° C, 10 minutes at 65º for poly (A) tailing reaction; 60 minutes at 16° C for ligation reaction; 15 minutes at 42° C, 5 minutes at 85º for reverse transcription reaction; and 5 minutes at 95° C, followed by 14 cycles of 95° C for 3 seconds, 60º C for 30 seconds; and a stop reaction at 99° C for 10 minutes for miR-Amp reaction. cDNA samples were stored at -70° C until use in qPCR assays.

### Gene expression in qPCR

Assays were performed using 5 target genes (miR-146a-5p, miR-155-5p, miR-21-5p, miR-29c-3p and miR-125b-5p). Gene expression of each sample was performed in duplicate by qPCR in a custom assay, produced by Applied Biosystems®. qPCR amplification mixtures contained 5 μL of 2X TaqMan® Fast Advanced Master Mix and 0.5 μL TaqMan® Advanced miRNA Assays (both Applied Biosystems®) for expression of each target and reference gene (Cel-miR-39). The characteristics of genes used as molecular markers, including Assay IDs are shown in Table 1. Template cDNA (2.5 μL) and RNase-free water (2 μL) were added to the mixture in a total volume of 10 μL. Reactions were performed using a StepOne^™^ Real-Time PCR Systems (Applied Biosystems®) using the following thermal profile in Fast mode: 95° C for 20 seconds, followed by 40 cycles at 95° C for 1 second and 60° C for 20 seconds. In each reaction a negative control was added (mix only). Negative results (without PCR amplification) were repeated until three times.

**Table 1:** miRNA gene expression analyses: Human target genes investigated in this study.

*Assay name* ^*a*^	*miR Base Accession number*	*Gene family*	*Assay ID* ^b^	*Chromosome location*	*Mature miRNA sequence*
hsa-miR-146a-5p	[MIMAT0000449]	MIPF0000103, mir-146	478399_miR	5	UGAGAACUGAAUUCCAUGGGUU
hsa-miR-155-5p	[MIMAT0000646]	MIPF00157, mir-155	477927_miR	21	UUAAUGCUAAUCGUGAUAGGGGU
hsa-miR-21-5p	[MIMAT0000076]	MIPF0000060, miR-21	477975_miR	17	UAGCUUAUCAGACUGAUGUUGA
hsa-miR-29c-3p	[MIMAT0000681]	MIPF0000009, miR-29	479229_miR	1	UAGCACCAUUUGAAAUCGGUUA
hsa-miR-125b-5p	[MIMAT0000446]	MIPF0000033, miR-125	477885_miR	11	UCCCUGAGACCCUAACUUGUGA
^c^ cel-miR-39-3p	[MIMAT0000010]	MIPF0000304, mir-39	478293_miR	ND ^d^	UCACCGGGUGUAAAUCAGCUUG

^a^ hsa, *Homo sapiens*

^b^ Purchased from Applied Biosystems

^c^ cel, *Caenorhabditis elegans*

^d^ Non-determinate

### Data analysis

miRNA levels (five genes) were expressed as “Relative Quantification” (RQ). qPCR amplification plots reflect the fluorescent signal in each cycle and were determined as threshold cycle (C_T_) values for each sample. The mean of C_T_ value was calculated after qPCR results (in duplicate) for each sample. C_T_ values were transformed into RQ by comparative C_T_ method (2-ΔΔC_T_) as described by Livak and Schimittgen [42]. miRNAs extracted from serum-derived EVs of negative individuals and CSF-derived EVs of OI/HIV patients were considerate as calibrators for calculations of miRNA expression. According to the comparative C_T_ method, the expression values of calibrators are considerate as 1.0.

Comparisons of EV concentration and EV size between groups were determined by Mann–Whitney test, one-tailed *p* value. In all cases, mean differences were considered statistically significant when *p* ⩽ 0.05 (* *p* <0.05, ** *p* <0.005, *** *p* <0.0005, **** *p* <0.00005). Statistical analyses were performed using Graph Pad Prism software version 6.0 (San Diego, CA, USA).

## Results

### Description of clinical samples

The clinical and laboratorial data of the 83 clinical samples used in this study are described in Table 2 and Table 3 that included: patient code, laboratorial diagnosis for toxoplasmosis (ELISA and qPCR), and clinical diagnosis. Table 2 describes group I, 20 negative sera for toxoplasmosis collected from healthy donors and healthy pregnant women; and group II, 21 sera from cerebral toxoplasmosis/HIV-coinfected patients and pregnant women with gestational toxoplasmosis. Table 3 describe group III, 26 CSF collected from CT/HIV patients; and group IV, 16 CSF collected from OI/HIV patients.

**Table 2:** Serum samples: Clinical and laboratory diagnoses of patients/individuals.

Number	Cod/Patient	ELISA	qPCR	Clinical diagnosis
1	3710/GJS	Neg	Neg	Healthy donor
2	3715/WFS	Neg	Neg	Healthy donor
3	3716/ARR	Neg	Neg	Pregnant
4	3753/HSC	Neg	Neg	Healthy pregnant
5	3754/AVO	Neg	Neg	Healthy pregnant
6	3778/PRS	Neg	Neg	Healthy pregnant
7	3809/IPT	Neg	Neg	Healthy pregnant
8	3843/JMRM	Neg	Neg	Healthy pregnant
9	3881/STM	Neg	Neg	Healthy pregnant
10	3958/MSO	Neg	Neg	Healthy pregnant
11	3991/JHVL	Neg	Neg	Healthy donor
12	50G/DS	Neg	ND	Healthy donor
13	01G/DS	Neg	ND	Healthy donor
14	63G/DS	Neg	ND	Healthy donor
15	65G/DS	Neg	ND	Healthy donor
16	68G/DS	Neg	ND	Healthy donor
17	70G/DS	Neg	ND	Healthy donor
18	74G/DS	Neg	ND	Healthy donor
19	75G/DS	Neg	ND	Healthy donor
20	399/DPL	Neg	Neg	Healthy pregnant
21	3621/JEC	Pos	Neg	Cerebral toxoplasmosis/AIDS
22	3622/WRR	Pos	Neg	Cerebral toxoplasmosis/AIDS
23	3623/RSL	Pos	Neg	Cerebral toxoplasmosis/AIDS
24	3624/JAS	Pos	Neg	Cerebral toxoplasmosis/AIDS
25	3625/CGC	Pos	Neg	Cerebral toxoplasmosis/AIDS
26	3626/EMAS	Pos	Pos	Cerebral toxoplasmosis/AIDS
27	3627/VM	Pos	Pos	Cerebral toxoplasmosis/AIDS
28	3628/HSC	Pos	Pos	Cerebral toxoplasmosis/AIDS
29	3918/WSO	Pos	Neg	Cerebral toxoplasmosis/AIDS
30	3830/RLN	Pos	Neg	Cerebral toxoplasmosis/AIDS
31	3717/MPG	Pos	Neg	Gestational toxoplasmosis
32	3850/JDR	Pos	Neg	Gestational toxoplasmosis
33	3910/RCP	Pos	Pos	Gestational toxoplasmosis
34	3923/KLV	Pos	Neg	Gestational toxoplasmosis
35	3924/AAZ	Pos	Neg	Gestational toxoplasmosis
36	3929/AAM	Pos	Neg	Gestational toxoplasmosis
37	3937/JCL	Pos	Neg	Gestational toxoplasmosis
38	3967/KRB	Pos	Neg	Gestational toxoplasmosis
39	3906/HRN	Pos	Neg	Gestational toxoplasmosis
40	3916/SGO	Pos	Neg	Gestational toxoplasmosis
41	3850/JDR	Pos	Neg	Gestational toxoplasmosis

ND, non-determined.

**Table 3:** CSF samples: Clinical and laboratory diagnoses of AIDS patients.

Number	Cod/Patient	ELISA	qPCR	Clinical diagnosis
1	3399/MBAA	Pos	Neg	Cerebral toxoplasmosis/AIDS
2	3452/MRS	Pos	Pos	Cerebral toxoplasmosis/AIDS
3	3491/DRP	Pos	Pos	Cerebral toxoplasmosis/AIDS
4	3495/MRP	Pos	Pos	Cerebral toxoplasmosis/AIDS
5	3501/MCS	Pos	Pos	Cerebral toxoplasmosis/AIDS
6	3505/JOM	Pos	Neg	Cerebral toxoplasmosis/AIDS
7	3518/MSP	Pos	Neg	Cerebral toxoplasmosis/AIDS
8	3523/JEC	Pos	Pos	Cerebral toxoplasmosis/AIDS
9	3540 /SAGL	Pos	Neg	Cerebral toxoplasmosis/AIDS
10	3559/ESG	Pos	Neg	Cerebral toxoplasmosis/AIDS
11	3598/JRS	Pos	Pos	Cerebral toxoplasmosis/AIDS
12	3607/FAB	Pos	Pos	Cerebral toxoplasmosis/AIDS
13	3608/JOM	Pos	Neg	Cerebral toxoplasmosis/AIDS
14	3613/RAM	Pos	Neg	Cerebral toxoplasmosis/AIDS
15	3720/WHP	Pos	Neg	Cerebral toxoplasmosis/AIDS
16	3928/GSR	Pos	Pos	Cerebral toxoplasmosis/AIDS
17	3932/VSF	Pos	Neg	Cerebral toxoplasmosis/AIDS
18	3955/DCS	Pos	Pos	Cerebral toxoplasmosis/AIDS
19	3961/VASS	Pos	Pos	Cerebral toxoplasmosis/AIDS
20	3978/LNSF	Pos	Pos	Cerebral toxoplasmosis/AIDS
21	3932/VSF	Pos	Pos	Cerebral toxoplasmosis/AIDS
22	3796/GMS	Pos	Pos	Cerebral toxoplasmosis/AIDS
23	3400/BCF	Pos	Neg	Cerebral toxoplasmosis/AIDS
24	3416/BCM	Pos	Neg	Cerebral toxoplasmosis/AIDS
25	3561/EMAS	Pos	Neg	Cerebral toxoplasmosis/AIDS
26	3724/HGM	Pos	Neg	Cerebral toxoplasmosis/AIDS
27	3728/CBA	Neg	Neg	Tuberculous meningitis/AIDS
28	3733/MENN	Neg	Neg	Tuberculous meningitis/AIDS
29	3737/FRCS	Neg	Neg	PML/AIDS^1^
30	3874/CAS	Neg	Neg	Tuberculous meningitis/AIDS
31	3888/ASS	Neg	Neg	Peripheral neuropathy/AIDS
32	3903/ABS	Neg	Neg	Herpes Zoster/AIDS
33	3908/FRG	Neg	Neg	Disseminated tuberculosis/AIDS
34	3015/FOV	Neg	Neg	Disseminated tuberculosis/AIDS
35	3922/EAG	Neg	Neg	Disseminated tuberculosis/AIDS
36	3936/EAS	Neg	Neg	Disseminated tuberculosis/AIDS
37	3953/FEG	Neg	Neg	Disseminated tuberculosis/AIDS
38	3964/AGS	Neg	Neg	Disseminated tuberculosis/AIDS
39	3965/DRG	Neg	Neg	PML/AIDS^1^
40	3972/FSS	Neg	Neg	HIV encephalitis/AIDS
41	3986/CB	Neg	Neg	Tuberculous meningitis/AIDS
42	4024/DJS	Neg	Neg	Pneumocystosis/syphilis/AIDS

^1^ PML, Progressive multifocal leukoencephalopathy.

### Characterization by NTA of serum-derived EVs and CSF-derived EVs of patients with toxoplasmosis

**Fig 1:**
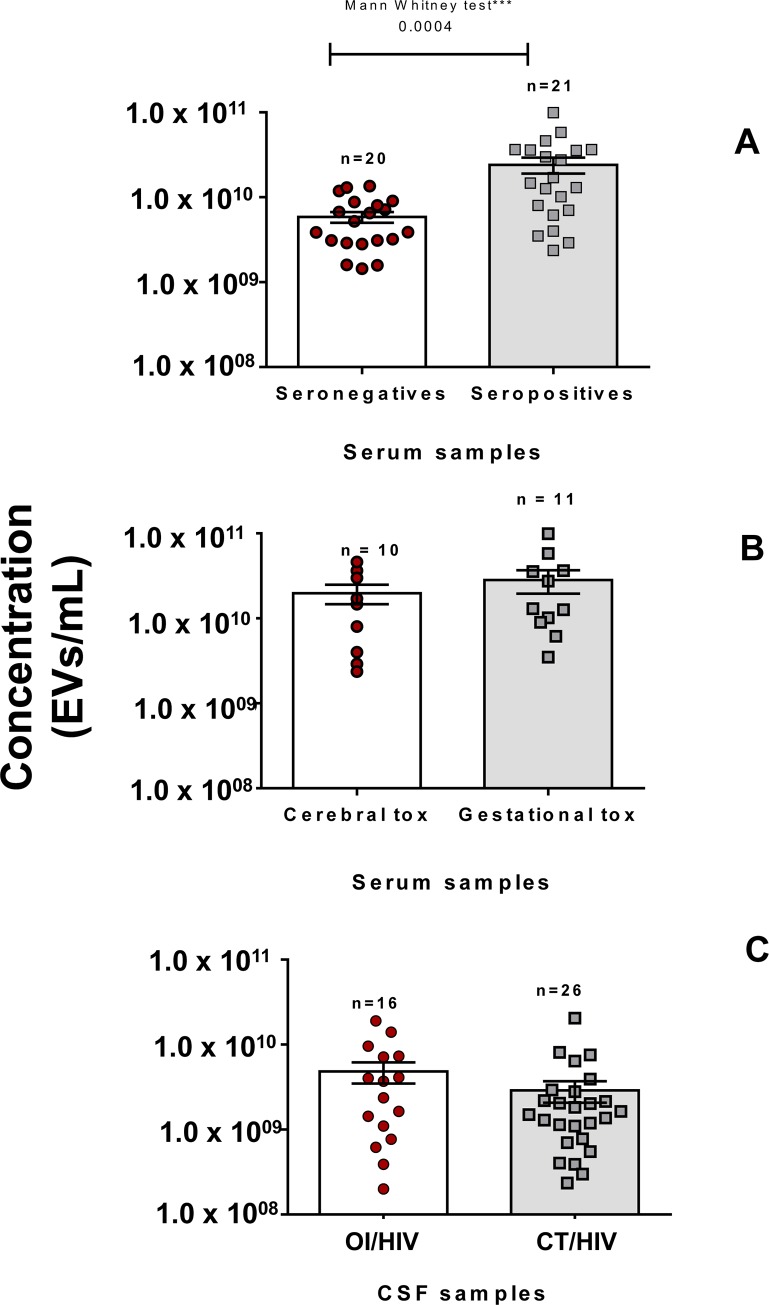
EVS evaluated by representative NTA. **(Panel A)**, distribution of concentration/mL of EVs purified by ultracentrifugation from 41 serum samples (250 μL) from seronegative individuals (red circles) and seropositive patients (gray squares). Differences in particle concentrations (EVs) between the two groups were statistically different at *** *p* = 0.0004 (Mann–Whitney test, one-tailed p value). **(Panel B)**, comparison between distribution of concentration (EVs/mL) from serum samples of cerebral toxoplasmosis patients (red circles) and pregnant women with gestational toxoplasmosis (gray squares). **(Panel C)**, distribution of concentration/mL of EVs purified by ultracentrifugation of 42 cerebrospinal fluid samples (500 μL) from AIDS patients with other opportunistic infections (OI/HIV) (red circles) and cerebral toxoplasmosis/HIV co-infection (CT/HIV) (gray squares).

After purification by ultracentrifugation, CSF derived EVs and serum-derived EVs were analyzed in NanoSight (size and concentration). Fig 1A shows the concentration of serum-derived EVs (250 μL) of 21 seropositive patients and 20 seronegative individuals for toxoplasmosis. Concentrations of serum-derived EVs of seropositive patients (mean: 2.4 x 10^10^ EVs/mL) were statistically higher than seronegative individuals (mean: 5.9 x 10^9^ EVs/mL).

The data produced by NTA were, also, used to compare the concentrations of serum-derived EVs of patients according clinical form of toxoplasmosis. Fig 1B shows concentrations of EVs of the 10 CT/HIV patients and 11 pregnant women with gestational toxoplasmosis. Among them, pregnant women had an increase in particle concentration (mean: 2.8 x 10^10^ EVs/mL) than CT/HIV patients (mean: 2.0 x 10^10^ EVs/mL), but without statistical difference.

Analyzes in CSF were distinct from sera ones, since is unusual to obtain CSF samples from health individuals. Thus, as criterion, CSF samples collected from patients with other opportunistic diseases and AIDS were used as control to compare with those from CT/HIV group. Fig 1C shows the concentration CSF-derived EVs (500 μL). Among them, the 26 CT/HIV patients had a decrease in particle concentration (mean: 2.9 x 10^9^ EVs/mL) than the 16 OI/HIV patients (mean: 4.8 x 10^9^ EVs/mL), but without statistical difference.

**Fig 2:**
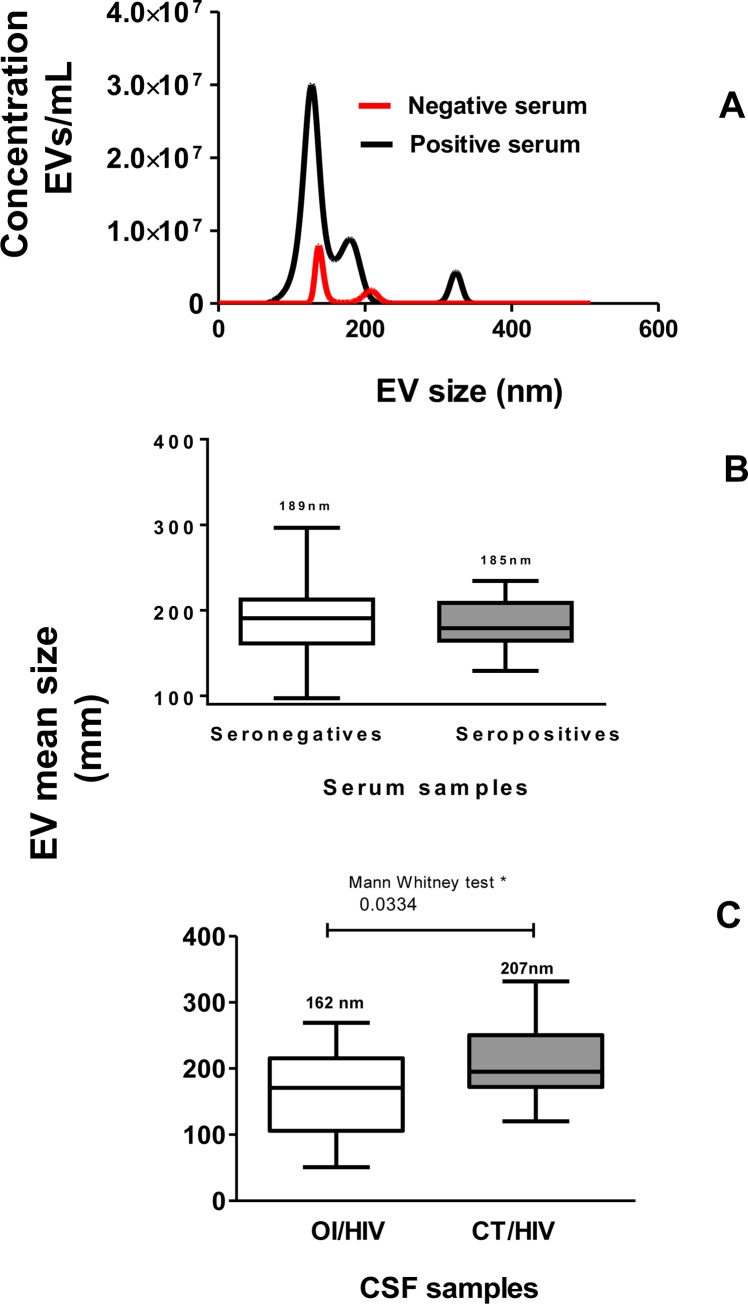
(Panel A), sera derived-EVs (seronegative—red line) and (seropositive—black line) were evaluated by representative size distribution (nm) and particles/mL in NanoSight equipment. Data represent three reads per sample. (Panel B), mean of EV size (in nm) purified by ultracentrifugation from 41 human sera. White column represents mean size of EVs purified from seronegative individuals and gray column, from seropositive patients. (Panel C), mean of EV size (in nm) purified by ultracentrifugation from 42 human CSF samples. White column represents mean size of EVs purified from OI/HIV patients and gray columns, from CT/HIV patients. In Panels C and D, values are represented by box and whiskers considering mean ±SEM.

In the next step was the particle size was investigated by NTA. Values represent the mean of three reads in NanoSight (concentration and size). Fig 2A shows the comparison between concentrations of serum-derived EVs from a seropositive patient and a seronegative individual. In addition, shows the similar mean size of EVs in both samples.

Regarding to size, Fig 2B shows the mean± SEM (standard error of the mean) size of serum-derived EVs, which was 189.0 ± 10.10 nm for negative and 185.3 ± 5.9 nm for positive sera. Fig 2C shows the mean± SEM size of CSF-derived EVs that was 206.9 ± 11.49 nm for OI/HIV patients. This value was statistically higher than OI/HIV patients, that was 162.8 ± 15.52 nm. These data show that a great part of both, serum-derived EVs and CSF-derived EVs were microvesicles.

### Electron microscopy and immunoblotting characterizing human EVs

**Fig 3:**
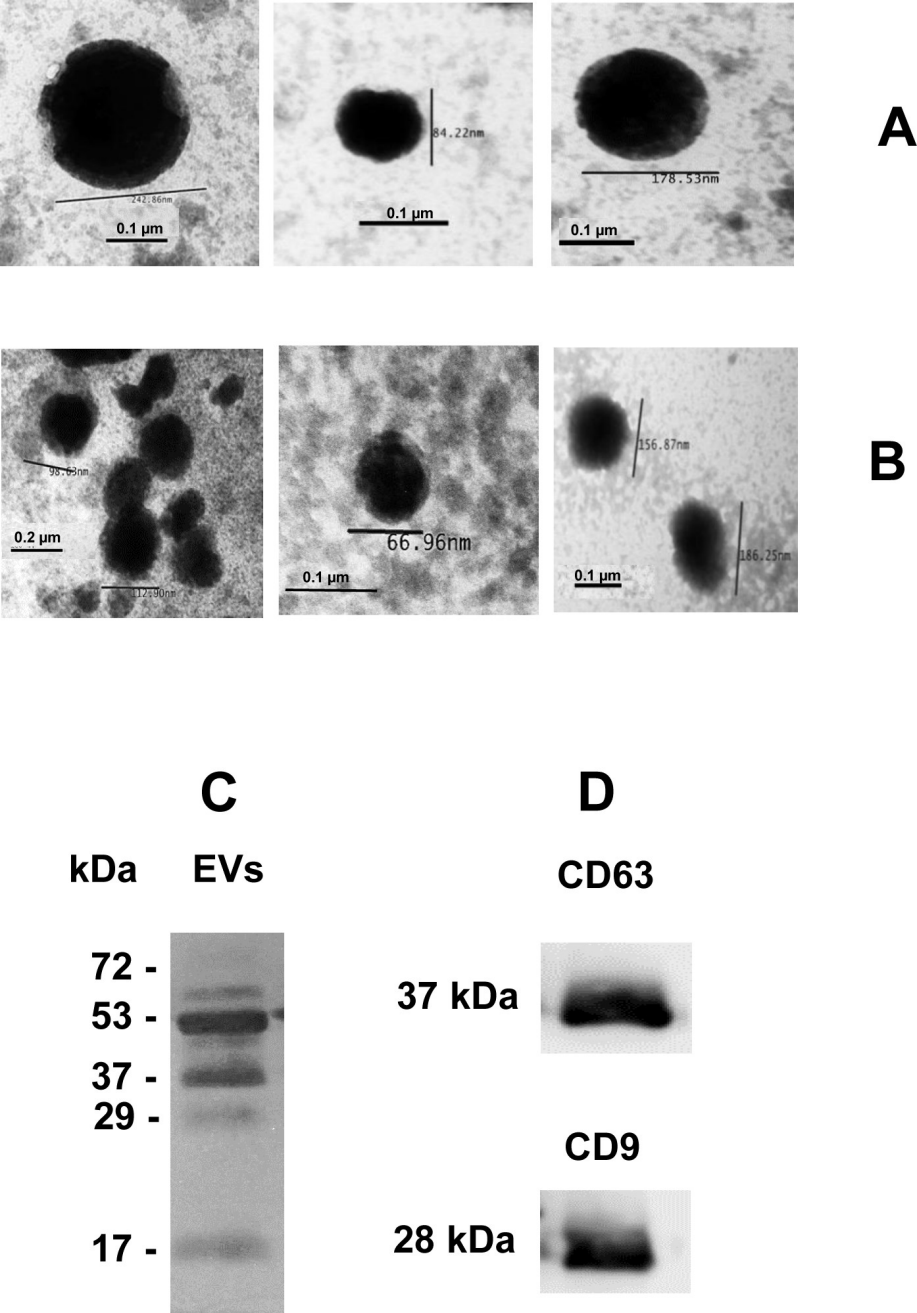
Images captured by transmission electron microscopy. Negatively stained microvesicles and exosomes of cerebrospinal fluid (**Panel A**) and serum (**Panel B**) purified by ultracentrifugation. Barr = 100 nm and 200 nm. Magnification of all images, 150, 000. Serum-derived EVs were separated by 10% SDS-PAGE and stained by silver. **(Panel C)**. Next, serum-derived EVs, separated by 10% SDS-PAGE was transferred to nitrocellulose and incubated with anti-CD63 and anti-CD9 antibodies (**Panel D)**.

NTA results were confirmed after analysis by TEM and immunoblotting. Fig 3A and Fig 3B show CSF-derived EVs and serum-derived EVs, respectively, after ultracentrifugation. The images reveal vesicles with typical size and shape of microvesicles and exosomes.

Microvesicles and exosomes have different transmembrane proteins. Exosomes have tetraspanins, including CD9 and CD63, which were detected by immunoblotting. Serum-derived EVs were separated by 10% SDS-PAGE and stained by silver (Fig 3C). After transference to nitrocellulose and incubation with anti-CD9 and anti-CD63 antibodies, the tetraspanins CD63, at 37 kDa, and CD9, at 28 kDa were reactive (Fig 3D). These results proved the presence of exosomes in preparations.

### Gene expression of miRNAs exosomal

**Fig 4:**
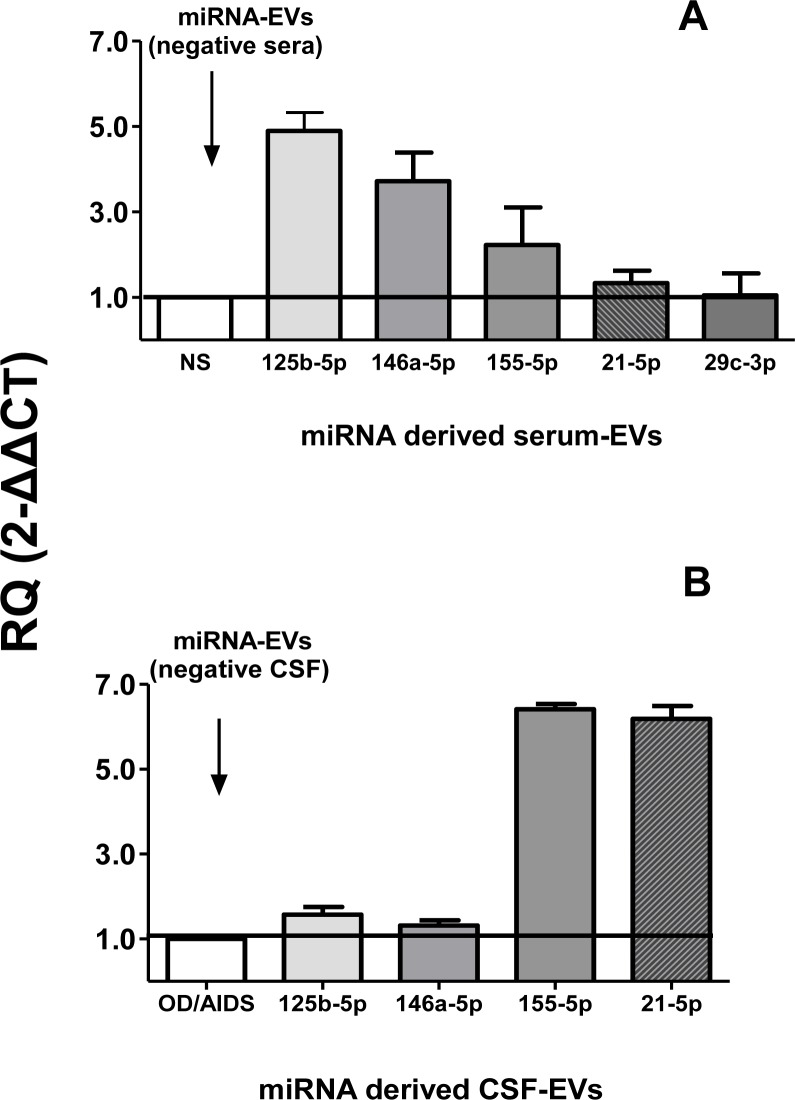
Relative expression of exosomal miR-125b-5p, miR-146a-5p, miR-155-5p, miR-21-5p and miR-29c-3p from serum-derived EVs **(Panel A)** and CSF-derived EVs **(Panel B)**. Assays were performed by qPCR. Values are expressed as mean ± SEM (standard error of the mean) of Relative Quantification (RQ) after calculation by comparative CT method (2-ΔΔCT) as described in Material and Methods section. The horizontal lines and write column indicate RQ values of calibrators (negative samples for toxoplasmosis), which were miRNAs from negative serum-derived EVs (NS) in **Panel A** and negative CSF-derived EVs (OI/HIV) in **Panel B**.

Next, the relative expressions of miR-146a-5p, miR-155-5p, miR-21-5p, miR-29c-3p and miR-125b-5p were estimated in miRNA purified from serum-derived EVs and CSF-derived EVs by qPCR. Fig 4 shows the relative expression of miRNA (expressed as RQ) extracted of serum-derived EVs (4A) and CSF-derived EVs (4B). RQ results of negative clinical samples (sera and CSF) for toxoplasmosis were considered as calibrators. Serum-derived EVs of group II, patients with gestational or cerebral toxoplasmosis up-expressed miR-125b-5p and miR-146a-5p. Values were 4.9 and 3.7 times higher than serum-derived EVs of healthy individuals (calibrator), respectively. However, for miR-155-5p and miR-21-5p, patients expressed 2.2 and 1.3 times higher than serum-derived EVs of healthy individuals, respectively. miR-29c-3p was similarly expressed in patients with toxoplasmosis and healthy individuals (Fig 4A).

CSF-derived EVs from CT/HIV patients up-expressed miR-155-5p and miR-21-5p, 6.4 and 6.2 times more than calibrators (EVs of OI/HIVP, respectively. miR-125b-5p (1.6 times) and miR-146a-5p (1.3 times) were similarly expressed in both groups of EVs. CSF-derived EVs were unable to express miR-29c-3p (Fig 4B).

## Discussion

Among EV functions produced in humans include the transference of different macromolecules and biomarkers interacting with specific molecules of immune system [20,21–23]. In *T*. *gondii* infection, parasite EVs stimulate the host immune system to produce host EVs (including microvesicles and exosomes). Infected host cells secret EVs with pathogen molecules able to induce modifications in uninfected cells or may serve as antigen presenters for the immune system [34]. At the same time, *T*. *gondii* EVs contain specific proteins as surface antigens, which are release by tachyzoites and compose the excreted-secreted antigens. These proteins participate of tachyzoite invasion, replication within host cells and are recognized by host immune system [29,31,43,44].

The evidence of exosomes/microvesicles in clinical samples includes: purification of EVs by ultracentrifugation; demonstration of exosomal proteins as tetraspanins; and presence of characteristic structures (in size and shape) by NTA and electron microscopy. For analyses of microvesicles and exosomes, purifications were performed in CSF and serum samples by ultracentrifugation. In this protocol, both types of EVs were recovered. Serum was chosen to minimize residual platelets that occur with standard plasma protocols [45,46].

As this article was the first that evaluated EVs purified from infected human with parasitic infection, this discussion was based in studies that described functions of human EVs produced in other diseases.

Concentrations of serum-derived EVs of seropositive patients were higher than those of non-infected individuals. Among those infected, pregnant women developing acute infection produced more EVs than CT/HIV patients, but without differences statistically significant. As the mean size of EVs was similar in both groups, these data suggested that only the concentration is able to distinguish patients with toxoplasmosis than healthy individuals. Similar results were shown in plasmas of patient with papillary thyroid carcinoma [47].

Analyzes of CSF-derived EVs were distinct from those of serum. As is unusual to obtain CSF from health individuals, CSF collected of patients with other opportunistic diseases and AIDS were used as negative control. Concentrations of CSF-derived EVs were almost similar in both groups, but lower than those of sera. Similar results were shown before [48,49].

EVs are produced also by neurons, microglia, and dendritic cells in the CNS [48]. Nevertheless, function of EVs in nervous system is not totally established. Studies have shown that EVs participate of the intercellular communication, maintenance of myelination, synaptic plasticity, antigen presentation, and trophic support of neurons [48–52]. Here, CSF samples of both patient groups, which were developing severe cerebral infection diseases, produced considerable concentration of EVs. According previous studies systemic inflammations caused by severe infections increase the release of EVs and these vesicles contain pro-inflammatory factors, including microRNAs, that directly cause the activation of cytokine production [48,50,51,53].

The investigation of particle size by NTA confirmed that EVs derived, serum and CSF had size and shape characteristic of microvesicles and exosomes. The average size of EVs was similar in serum and CSF groups. The majority of particles were microvesicles in sera (180–290 nm) and in CSF (162–300 nm). Presence of exosomes was also confirmed by size and shape in TEM and the evidence of the tetraspanins CD63 and CD9 confirmed in immunoblotting after SDS-PAGE.

Small RNAs participate in transcriptional gene regulation in cell development and other biological processes such as cell differentiation, proliferation and apoptosis [54]. Among small RNAs, miRNAs, which can be transported by EVs are regulatory elements that can modulate gene expression at the post-transcriptional level [24]. miRNAs, yet may be important in controlling Th1 differentiation, and to produce increased levels of IFN-γ, implicating miRNAs in the regulation of Th1 response [24,54].

HIV/AIDS patients with low number of CD4 normally develop opportunistic infections. At the same time, EVs can cause inflammations in HIV/AIDS patients via host miRNAs and viral miRNAs [54,55]. However, until now is unknown the participation of the miRNAs in different co-infection caused by HIV and other pathogens. Patients developing all toxoplasmosis forms had circulating tachyzoites, IFN-γ inhibition and high TNF-α production. This inflammatory response contributes for damage of the choroid and retina and encephalitis [17,56].

Serum-derived EVs from CT/HIV patients and pregnant women, with acute toxoplasmosis up-expressed miR-125b-5p and miR-146a-5p, when compared with serum-derived EVs of healthy individuals. Elevation of TNF-α, IL-6, and downregulation of IFN-γ, as already reported in AIDS patients, marked upregulation of miRNA 125b-5p [53,57]. In the same way, miR-146a-containing exosomes reduce cytokine expression, as IFN-γ, IL-17 and IL-2 [57–59].

Nevertheless, CSF-derived EVs of both groups of patients, CT/HIV and OI/HIV expressed equality miR-125b-5p, and miR-146a-5p. CSF-derived EVs of CT/HIV patients compared with those of OI/HIV patients up-expressed miR-155-5p and miR-21-5p. These data can suggest the induction of pro-inflammatory cytokine expression caused by miR-155 in CT/HIV co-infection that was more intense than those from other co-infections analyzed here. At the same time, the up-expression of miR-21-5p suggested the impact on the balance between Th1 and Th2 [58,59].

miR-21-5p expression by serum-derived EVs was almost similar in both patient groups (with or without toxoplasmosis). Similar results were observed by Akers et al. [26] when studied EVs from glioblastoma patients. The authors demonstrated that miR-21-5p was more expressed in CSF-derived EVs than those of serum. On the other hand, Lakhter et al. [60] have shown that exosomal miR-21-5p may be up-expressed in the presence of IFN-γ and TNF-α.

Serum-derived EVs in patients with toxoplasmosis down-expressed miR-29c-3p. CSF-derived EVs were unable to express miR-29c-3p. IFN-γ has a critical role in the immune response in intracellular infections and miR29c-3p participates as a regulator of Th1 and IFN-γ in innate and adaptive immunity. According other authors [57,61,62], down or non-expression of miR29c-3p can cause an increase in IFNγ. However, the data here presented could suggest that EVs produced in AIDS patients, other miRNAs may play more significant role in suppressing IFN-γ.

Finally, all these data together demonstrate the presence of microvesicles and exosomes expressing exosomal miRNAs in patients with cerebral or gestational toxoplasmosis Exosomes containing miRNA can participate in modulation of cellular response. Results demonstrated the presence of a heterogeneous population of EVs in serum and CSF samples.

## Supporting information

S1 Data Comp soro neg e pos humano2. (ZIP)

S2 Data Comparison concentr EVs in human serum1. (ZIP)

S3 Data Concentr EVs in human CSF1. (ZIP)

S4 Data Concentr EVs in human serum1. (ZIP)

S5 Data CSF—All miRNAs4. (ZIP)

S6 Data Sera—All miRNAs4. (ZIP)

S7 Data Size human EVs in CSF—Mean2. (ZIP)

S8 Data Size human EVs in serum—Mean2. (ZIP)

S1 File (PDF)

S1 Fig (PPTM)

S2 Fig (PPTM)

S1 Results (XLSX)
