# Supplementary material for: Human extracellular vesicles and correlation with two clinical forms of toxoplasmosis
Source: PLoS One. 2020 Mar 3;15(3):e0229602. doi: 10.1371/journal.pone.0229602 (PMC7054008; doi:10.1371/journal.pone.0229602)
Supplement: S1 File — (PDF) [file pone.0229602.s009.pdf]

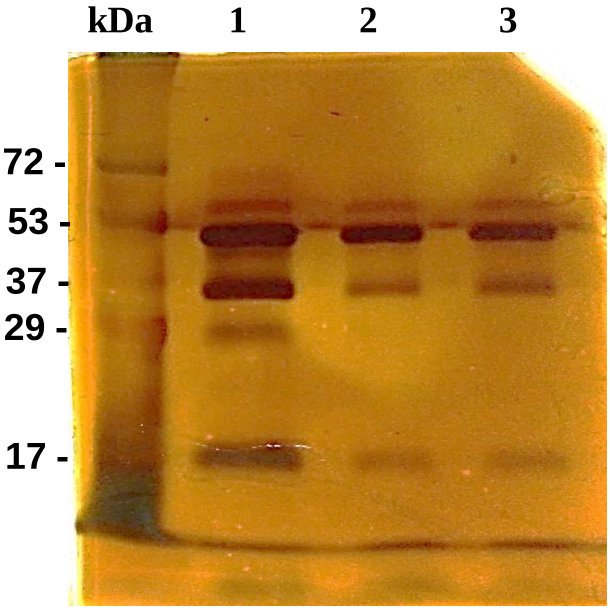

Electrophorese - Serum-derived EVs separated by 10% SDS-PAGE and stained by silver.
Three samples

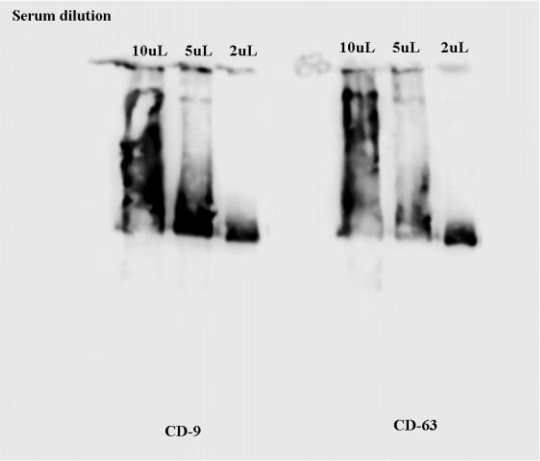

Immunoblot:
serum-derived EVs, separated by 10% SDS-PAGE, transferred to nitrocellulose and incubated with anti-CD63 and anti-CD9 antibodies.
Three concentrations were used for standardization.
