# Supplementary material for: Human extracellular vesicles and correlation with two clinical forms of toxoplasmosis
Source: PLoS One. 2020 Mar 3;15(3):e0229602. doi: 10.1371/journal.pone.0229602 (PMC7054008; doi:10.1371/journal.pone.0229602)
Supplement: S1 Fig — (PPTM) [file pone.0229602.s010.pptm]

## Slide 1
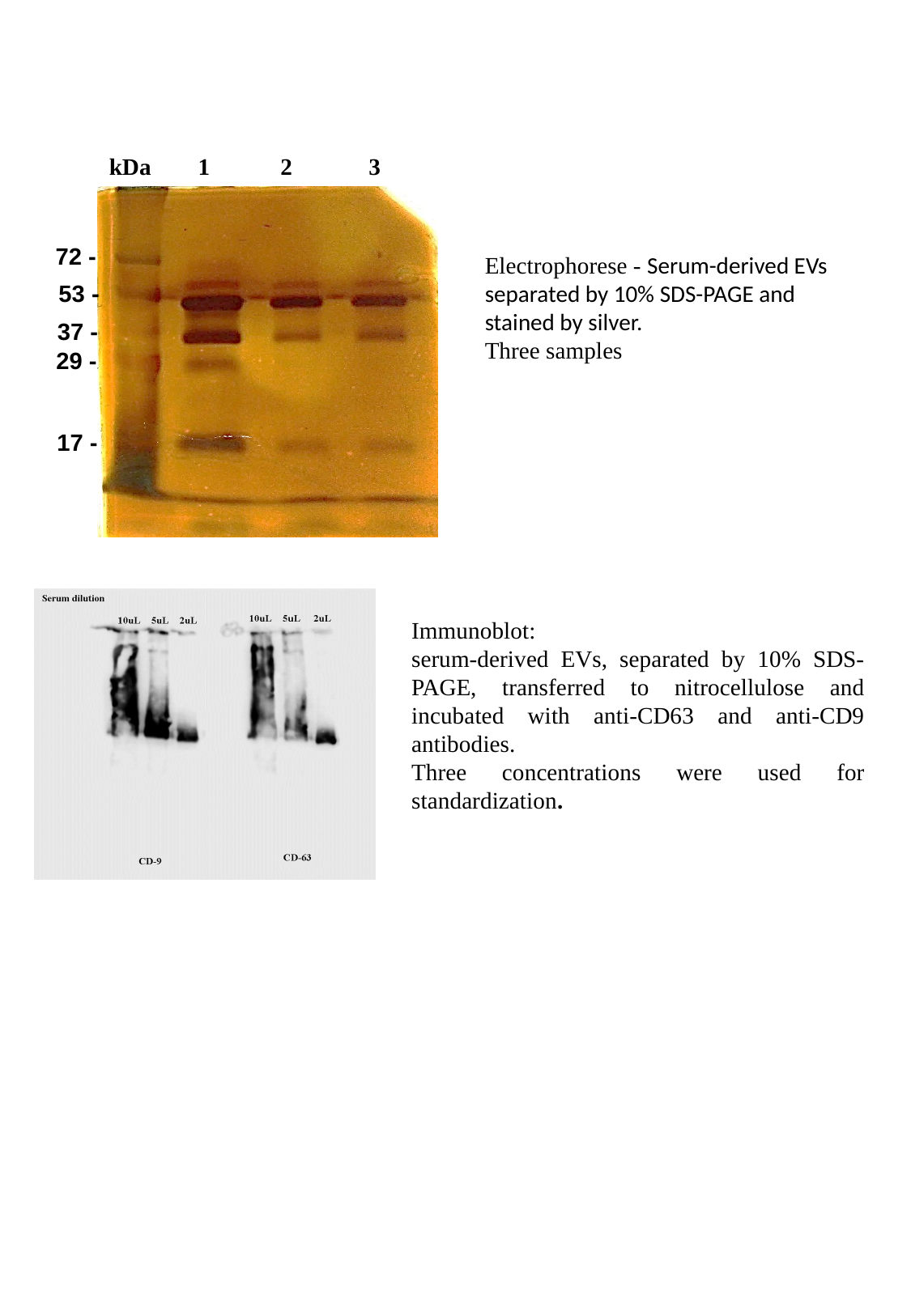

kDa 1 2 3
72 -
53 -
37 -
29 -
17 -
Electrophorese - Serum-derived EVs separated by 10% SDS-PAGE and stained by silver.
Three samples
Immunoblot:
serum-derived EVs, separated by 10% SDS-PAGE, transferred to nitrocellulose and incubated with anti-CD63 and anti-CD9 antibodies.
Three concentrations were used for standardization.
